# Risk of Reactivation of Hepatitis B in Hepatitis B Surface Antigen-Negative and Hepatitis B Core Antigen Antibody Positive Patients Receiving Biologic Therapy

**DOI:** 10.5152/tjg.2023.23070

**Published:** 2023-03-01

**Authors:** Zeynep Gök Sargın

**Affiliations:** Department of Gastroenterology and Hepatology, Kırıkkale University Faculty of Medicine, Kırıkkale, Turkey

Dear Editor,

The journal’s online paper by Ergenç et al^1^ entitled “Biologic Therapy Carries a Very Low Risk of Reactivation in Hepatitis B Surface Antigen-Negative Phase of Hepatitis B” caught our attention. The article is investigating the necessity of prophylactic anti-hepatitis B virus (HBV) therapy for all biologic agents in hepatitis B surface antigen (HBsAg)-negative and hepatitis B core antigen antibodies (anti-HBc) immunoglobulin G (IgG)-positive patients with immune-mediated inflammatory diseases. It is also supported by real-world data showing low reactivation risk compared to existing studies. We would like to congratulate the authors for analyzing such a large amount of data and highlighting a clinically controversial issue; however, some points merit discussion.

The authors stated that almost 80% of their patients did not receive antiviral prophylaxis.^1^ In isolated anti-HBc IgG-positive patients with a low or moderate risk of HBV reactivation, pre-emptive therapy may be preferred. This includes assessing HBsAg and HBV deoxyribonucleic acid (DNA) every 3 months during and after cessation of immunosuppression and beginning antiviral treatment in case either of these markers becomes detectable.^2^ Isolated anti-HBc IgG-positive patients with a negative HBV DNA should undergo liver tests every 1 to 3 months and HBV DNA at a 3-month interval until 6 to 12 months after immunosuppressive therapy.^2–4^ The unavailability of follow-up information is a limitation of this study, as 9.5% of patients did not have systematic monitoring. This study’s lower reactivation rate might be due to unnoticed asymptomatic reactivation.

We would also like to emphasize that the possibility of occult hepatitis B infection has been ignored in this cohort. As stated by the authors, approximately 75% of the patients in this study were not tested for HBV DNA before receiving biological therapies. According to the European Association for the Study of the Liver (EASL) and American Association for the Study of the Liver Diseases (AASLD) guidelines, it is necessary for HBsAg, anti-HBc IgG, and anti-HBs screening before starting any immunosuppressive therapy in countries where the prevalence of HBsAg is more than 2%. Hepatitis B virus DNA must be tested in case of HBsAg and/or anti-HBc positivity.^2–4^ Occult hepatitis B infection refers to the existence of viral replication in the liver of patients with a negative HBsAg test. The frequency of occult hepatitis B differs considerably among various populations. Asia and West Africa have higher prevalence rates.^5^ Turkey, in the intermediate endemic region, has a 4% positivity rate for HBsAg and a 30.67% anti-HBc positivity rate. Hepatitis B virus exposure affects roughly one-third of the adult population, meaning these people have the virus’ covalently closed circular DNA (cccDNA) in their livers.^4^ For this reason, ignoring the possibility of occult hepatitis B infection in patients whose HBV DNA was not tested before biological therapy was another limitation of this study.
